# Assessing the effectiveness of enhanced psychological care for patients with depressive symptoms attending cardiac rehabilitation compared with treatment as usual (CADENCE): study protocol for a pilot cluster randomised controlled trial

**DOI:** 10.1186/s13063-016-1184-9

**Published:** 2016-02-02

**Authors:** Suzanne H. Richards, Chris Dickens, Rob Anderson, David A. Richards, Rod S. Taylor, Obioha C. Ukoumunne, David Kessler, Katrina Turner, Willem Kuyken, Manish Gandhi, Luke Knight, Andrew Gibson, Antoinette Davey, Fiona Warren, Rachel Winder, Christine Wright, John Campbell

**Affiliations:** University of Exeter Medical School, St Luke’s Campus, Exeter, EX1 2LU UK; NIHR Collaboration for Leadership in Applied Health Research and Care for the South West Peninsula, University of Exeter Medical School, St Luke’s Campus, Exeter, EX1 2LU UK; School of Social and Community Medicine, University of Bristol, Oakfield House, Oakfield Grove, Clifton, Bristol, BS8 2BN UK; School of Social and Community Medicine, University of Bristol, Canynge Hall, Whatley Road, Bristol, BS8 2PS UK; University Department of Psychiatry, University of Oxford, Warneford Hospital, Oxford, 0X3 7JX UK; Royal Devon and Exeter NHS Foundation Trust, Barrack Road, Exeter, EX2 5DW UK; Health and Social Sciences, University of the West of England, Frenchay Campus, Coldharbour Lane, Bristol, BS16 1QY UK

**Keywords:** Depression, Coronary heart disease, Multimorbidity, Behavioural activation, Mental health care coordination, Cardiac rehabilitation, Randomised controlled trial, Qualitative interviews

## Abstract

**Background:**

Around 17 % of people eligible for UK cardiac rehabilitation programmes following an acute coronary syndrome report moderate or severe depressive symptoms. While maximising psychological health is a core goal of cardiac rehabilitation, psychological care can be fragmented and patchy. This study tests the feasibility and acceptability of embedding enhanced psychological care, composed of two management strategies of proven effectiveness in other settings (nurse-led mental health care coordination and behavioural activation), within the cardiac rehabilitation care pathway.

**Methods/Design:**

This study tests the uncertainties associated with a large-scale evaluation by conducting an external pilot trial with a nested qualitative study. We aim to recruit and randomise eight comprehensive cardiac rehabilitation teams (clusters) to intervention (embedding enhanced psychological care into routine cardiac rehabilitation programmes) or control (routine cardiac rehabilitation programmes alone) arms. Up to 64 patients (eight per team) identified with depressive symptoms upon initial assessment by the cardiac rehabilitation team will be recruited, and study measures will be administered at baseline (before starting rehabilitation) and at 5 months and 8 months post baseline. Outcomes include depressive symptoms, cardiac mortality and morbidity, anxiety, health-related quality of life and service resource use. Trial data on cardiac team and patient recruitment, and the retention and flow of patients through treatment will be used to assess intervention feasibility and acceptability. Qualitative interviews will be undertaken to explore trial participants’ and cardiac rehabilitation nurses’ views and experiences of the trial methods and intervention, and to identify reasons why patients declined to take part in the trial. Outcome data will inform a sample size calculation for a definitive trial.

**Discussion:**

The pilot trial and qualitative study will inform the design of a fully powered cluster randomised controlled trial to evaluate the effectiveness and cost-effectiveness of the provision of enhanced psychological care within cardiac rehabilitation programmes.

**Trial registration:**

ISRCTN34701576 (Registered 29 May 2014)

**Electronic supplementary material:**

The online version of this article (doi:10.1186/s13063-016-1184-9) contains supplementary material, which is available to authorized users.

## Background

### Prevalence of depression and associated outcomes for patients with acute coronary syndromes

Major depression is common among people with coronary heart disease. Most research has focused on patients with acute coronary syndromes (ACS), among whom prevalence rates for depression are around 20 % as determined by studies using rigorous clinically structured research interviews [1]. Rates of depression have also been shown to be elevated in individuals following coronary artery bypass grafting [2], patients with unstable angina [3] and patients with chronic heart failure [4]. These rates greatly exceed those seen in the UK general population (2.6 % [5]), suggesting that the associations between coronary disease and depression may be causal (direct or indirect).

The nature of the association between depression and coronary disease is complex. Depression can predate an ACS, approximately doubling the risk of subsequent incident ACS [6, 7], and worsen associated heart failure [8] or cardiac mortality [9]. Depression that predates an ACS is predicted by younger age, female sex, lack of social support, ongoing life difficulties and a past psychiatric history [10]; these are similar to risk factors for depression in the general population. Depressive symptoms may occur after an ACS, so-called ‘new onset’ depression [10]; approximately 20 % of individuals after a recent myocardial infarct (MI) develop depressive symptoms in the 12 months following their cardiac event. ‘New onset’ depression is not associated with the usual risks for depression, but is predicted by negative illness representations relating to the ACS and ongoing cardiac symptoms [10, 11]. Mechanisms underpinning the association between depressive symptoms and poor cardiovascular outcomes may include biological and behavioural processes, or may be confounded by shared genetic vulnerability, environmental stresses, or perseverative negative cognitive processes [12].

Depression among people with an established ACS is an important predictor of negative medical outcomes including poorer health-related quality of life [13, 14], greater morbidity and mortality [15–18], greater use of routine and unscheduled health care [19, 20] and greater health care costs [21]. Some studies have indicated that the timing of onset of depressive symptoms with regards to cardiac-related hospitalisation may be important in determining health outcomes. In particular, ‘new onset’ depression that develops following an MI has the greatest association with subsequent mortality [21–24]. It remains unclear, however, whether ‘new onset’ depression is particularly ‘cardiotoxic’ or whether its apparent associations with poor cardiac outcomes is confounded [23] by, for example, the severity of the underlying cardiac disease or due to a retention bias, with patients suffering with premorbid depression dying before reaching follow-up [9].

### Usual care for patients with cardiac disease and depression

**Fig. 1:**
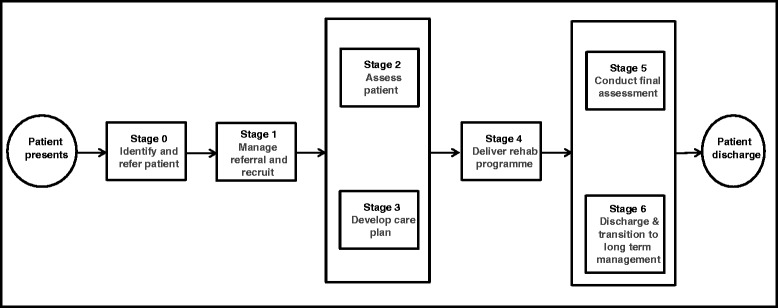
UK cardiac rehabilitation care pathway. UK Department of Health’s ‘0–6 Stage Patient Pathway of Care’ for cardiac rehabilitation services [83]

In the UK, the NHS routinely offers multidisciplinary cardiac rehabilitation (CR) to patients who experience an acute cardiac event. CR follows a seven-stage standard patient care pathway (stages 0–6, Fig. 1) [25, 26]. Guidance on the recommended content of standard care is published by the British Association for Cardiovascular Prevention and Rehabilitation (BACPR) [26]. Core components include: education; exercise and physical activity; diet and weight management; medical management; and psychological support. In 2010–2011 a national CR audit [27] identified 290 operationally distinct, locality-based comprehensive cardiac rehabilitation programmes (CCRPs; stages 2–5). These teams undertake patient assessments (including mental health status), then generate and implement CR care plans.

Patient attrition across the CR pathway is high. Although around 17 % of patients screen positive for depressive symptoms when attending an initial, locality-based CCRP assessment (stages 2–3), around half of these will not proceed to CCRP. Despite the burden of depressive symptoms, only 10 % of CR services report direct psychology input [27] and patients’ depression usually remains untreated [28]. Locally agreed referral protocols providing for access to psychological care at either the tertiary (stage 1) or community (stages 2–5) level may be agreed. However, the precise content of such protocols and the consistency of their implementation remain unclear from audit data.

While it would appear that structured management of depressive symptoms is not routinely provided in CR, patients can access mental health care through existing care pathways for depression [29–31]. Effective care includes mental health care coordination, which is often managed in primary care and involves the use of stepped care algorithms, and access to psychological or pharmacological interventions of proven effectiveness.

In general populations, cognitive behavioural therapy (CBT) and antidepressant medication (ADM) are the two treatments with most evidence of effectiveness, and both are recommended by the National Institute for Clinical Excellence (NICE) [29, 30]. However, there are problems with ADM, which include side-effects, poor patient adherence and relapse risk on ADM discontinuation. Service-user organisations and policy think tanks advocate greater availability of psychological therapies, which many people prefer [32]. CBT, which is of similar efficacy to ADM [33], has several advantages: it is consistent with many service users’ preferences for non-pharmacological treatment, and it modifies the illness trajectory as its benefits continue after the end of treatment by teaching skills to prevent depressive relapse in the long term. However, CBT has several potential disadvantages. Not all patients may engage or adhere to CBT, due to a multiplicity of reasons (e.g. burden of homework, feelings of confusion or upset at revisiting painful or difficult emotions and situations during therapy) [34]. CBT requires extensive training to deliver competently, and is typically offered only by specialist CBT therapists. The high costs of training and employing sufficient therapists may limit attempts to access CBT. The recent ‘Improving Access to Psychological Therapies’ (IAPT) [35, 36] programme in England has done much to enhance access and recovery rates which are at approximately 50 %. However, IAPT does not reach into specialist CR settings where brief and accessible psychological treatments may have much to offer.

One such psychological treatment is that of Behavioural Activation (BA). BA is postulated to alleviate depression by focusing directly on changing behaviour based on behavioural theory [37]. This theory states that depression is maintained by avoidance of normal activities. As people withdraw and disrupt their basic routines, they become isolated from positive reinforcement opportunities in their environment. They may then end up stuck in a cycle of depressed mood, decreased activity and avoidance [37]. BA systematically disrupts this cycle, initiating action in the presence of negative mood, when people’s natural tendency is to withdraw or avoid [38]. Although CBT incorporates some behavioural elements, these focus on initiating behavioural experiments to test specific beliefs. In contrast, BA targets avoidance from a contextual and functional approach not found in CBT, focusing on understanding the function of behaviour and replacing it accordingly. BA also explicitly prioritises the treatment of negatively reinforced avoidance and rumination. Furthermore, the BA rationale is easier to understand and operationalise for both patients and mental health workers than CBT, where activity is also increased but the primary techniques focus on changing maladaptive beliefs [39]. Its focus on context and functioning is also closely aligned to the ethos of CR services. The relative simplicity of BA treatment makes it relatively straightforward to train nurses without mental health training in its application [40–42]. The study team includes the expertise in respect of the design, training, and delivery of a BA-based intervention [41–43].

### Rationale for current research

There is a lack of clear evidence of efficacy for conventional treatments for depression in patients with ACS. Trials of the antidepressants sertraline, citalopram and mirtazapine have shown small or no effect on depression among patients with ACS [44–46]. Efficacy of psychological treatments, which are usually preferred by patients, remains unclear. Recent systematic reviews [47, 48] report modest but significant reductions in depression for patients with coronary disease receiving psychological treatment, although the methodological quality of studies selected is such that it precludes firm conclusions regarding which treatments are most effective. Several high-quality randomised controlled trials (RCTs) in patients with ACS and depression have not confirmed the benefits of psychological therapy in this population. In the large ENRICHD trial, CBT plus citalopram led to very small improvements in depression that were not sustained [49]. Similarly, the CREATE trial found that interpersonal psychotherapy was no better than standard care [44]. In contrast, the CODIACS Vanguard trial RCT [50, 51] found that a patient preference intervention (management options available included problem-solving therapy delivered by a centralised service offered via phone or the web, ADM, both or neither) combined with a stepped care algorithm significantly improved depressive symptoms at 6-months post randomisation. However, these results must be interpreted with caution as the sample size was small (*n* = 150), and no conclusions can be drawn regarding the effectiveness of individual therapeutic options available as part of the preference intervention. No trials to date have evaluated the effectiveness of BA in patients with an ACS and ‘new onset’ depression.

Most intervention studies have recruited participants immediately following an acute cardiac event, and the complexity of depression in that period may have contributed to the lack of treatment response. The reasons for the limited benefits of conventional depression treatments in patients with coronary disease are not clear. First, depression that starts after an ACS is different from depression in the general population. Post-ACS depression does not have the usual risk factors as seen in the general population, but is associated with ongoing cardiac symptoms, and increased concerns about health [10, 11], which may act to reduce the response to conventional treatment [45]. Second, conventional psychological treatments, such as CBT, that encourage recall of past experience and that challenge maladaptive thoughts, may be too traumatic for people who have suffered a recent, serious threat to life. Third, conventional psychological therapies available through IAPT are considered expensive and may not be widely available to patients with CHD. Indeed, a recent qualitative study of patients [52] concluded that management of depression should be embedded within CR teams rather than as another source of referral. UK CR programmes experience significant patient attrition at each stage of the rehabilitation pathway, with over half of patients dropping out before locality-based CCRP commences. Given this attrition, for the 17 % of patients with depressive symptoms who actually attend at least one assessment with a CCRP, ‘another outward referral’ for psychological care may simply be a further barrier to accessing timely care. If a low-intensity psychological treatment, such as BA, has the potential to be safely and effectively [38, 40–42, 53, 54] embedded within the CCRP, this could holistically tackle the depressive symptoms at the same time as physical rehabilitation as part of patient case management. Similarly, acknowledging that attrition from rehabilitation is high even in patients who attend an initial CCRP assessment, we also believe that any attempt to enhance psychological care should include training CR nurses (who routinely screen for depressive symptoms) to apply evidence-based referrals for those who elect not to attend rehabilitation. Such mental health care coordination will also be in place for patients completing CCRP (including BA), but whose symptoms do not respond.

### Study aims and objectives

The overarching aim of this pilot study is to test the methods and procedures required to undertake a fully powered evaluation of the clinical effectiveness and cost-effectiveness of CR teams implementing an enhanced psychological care intervention (EPC) for patients with ‘new onset’ depressive symptoms using CR services compared with CR services delivering usual care. Specific objectives are:

1. To quantify the flow of patients (i.e. eligibility, recruitment and attrition rates) from the cardiac event to the 8-month follow-up for patients entering the CR care pathway, and in particular, to document the flow of those patients who agree to take part in the pilot trial
2. To collect participant outcome data in order to estimate the standard deviation for continuous outcomes to inform sample size calculations for a definitive trial
3. To establish the data collection methods required to support a definitive economic evaluation
4. To gather qualitative evidence from patients (including participants who did or did not adhere to the EPC, and those who declined to take part in the trial) and CR nurses on the acceptability of EPC, and on the appropriateness of study methods and procedures. Information will also be sought on the content of usual psychological care within CR teams

## Methods/Design

Consistent with Medical Research Council (MRC) guidance [55] a two-phase study was planned, including a mixed-method feasibility study, followed by a pilot cluster randomised controlled trial (RCT) and nested qualitative interview study.

### Pre-pilot feasibility study

Although the focus of this paper is to report the design of the pilot trial, a brief summary of the feasibility study is provided here. The feasibility study (completed March 2015 prior to commencing the pilot trial) employed a mixed-method design to develop and assess an EPC intervention and to undertake preliminary testing of its implementation. Observations of usual care, a before and after observational study, and qualitative interviews with staff and service users were undertaken. The EPC intervention was developed working with four nurses from three NHS CCRP services (stages 2–5; see Fig. 1) from South West England. Nine eligible patients (i.e. individuals with ‘new onset’ depression) were recruited, and both staff and patients were invited to comment on the feasibility and acceptability of implementing and/or experiencing EPC. Additional CR process data was recorded, including participant attendance at, and adherence with the CCRP with embedded EPC. The findings from this feasibility study were used to refine and develop the EPC intervention and the pilot trial methods. Specifically, qualitative and observational data, considered along with findings arising from discussions with lay representatives, identified the need to reduce the intensity of the BA component of the EPC delivered by CR nurses operating within the context of routine care. The EPC intervention to be delivered in the pilot study will thus retain the nurse acting as a mental health care coordinator, using clearly defined clinical decision-making points and applying evidence-based referral pathways, with the BA component delivered as a participant-led BA intervention, actively supported by the CR nurse.

### Pilot cluster randomised controlled trial

**Fig. 2:**
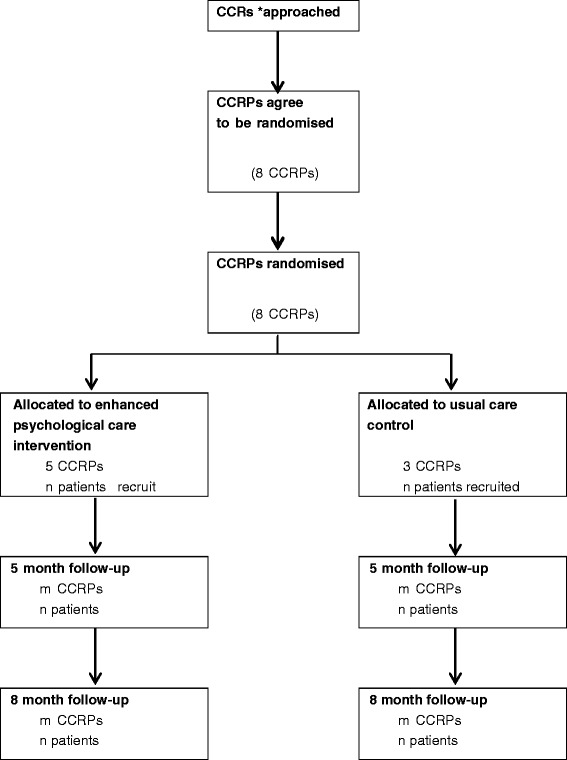
Consolidated Standards of Reporting Trials (CONSORT) diagram. CONSORT diagram (with cluster extension) for the CADENCE pilot randomised controlled trial (RCT)

An external pilot cluster RCT [56, 57] will be undertaken, including the piloting of economic data collection, and a qualitative interview study. A Consolidated Standards of Reporting Trials (CONSORT) diagram describing the flow of participants through the study is presented in Fig. 2.

### Trial interventions

#### Usual care (control) intervention

Usual care is defined here as the standard CR rehabilitation commencing at the point of assessment undertaken by a CR specialist nurse from a locality-based CCRP (stages 2–3). Of patients invited to attend CCRP, around 41 % attend structured sessions within 2–10 weeks (stage 4) [27]. Treatment as usual in CCRPs typically includes intensive rehabilitation for 1–2 sessions per week for approximately 8 weeks. Sessions generally last around 2 hours and include structured exercise, education (e.g. managing lifestyle and cardiac risk) and some psychological input (e.g. relaxation, stress management) in order to meet the core standards of care described by BACPR [26]. On exiting CCRP, a final assessment (stage 5) and discharge arrangements will be made to community services (stage 6).

There is debate as to what constitutes standard psychological care within CR [27], and a core objective is to clarify this definition. National audit data found that psychological expertise within locality-based CCRPs is uncommon (< 10 %). In the feasibility study, some patients with depressive symptoms were referred to a clinical psychologist. However, no systematic approach was routinely adopted to identify patients as being suitable for referral. The decision was pragmatic, being based on a combination of reviewing depression scores obtained in standard CCRP assessments, discussions with patients, and clinical judgment regarding who might benefit from referral. Informal discussions with mental health specialists and CR staff suggest that there is considerable variation in local CCRP protocols across the UK.

#### Enhanced psychological care (treatment) intervention

Based on our feasibility findings, we propose to embed EPC within existing CR care pathways, with referrals to existing community mental health services as appropriate. CR nurses from operationally distinct ‘clusters’ – locality-based CCRPs (see Fig. 1, stages 2–5) will be trained to deliver EPC based on a nurse training manual developed and refined during the feasibility study. The CR nurse will implement mental health care coordination [29, 30], including an embedded participant-led BA programme [38, 40–42, 53, 54]. Pre-existing participant and nurse training materials used by the co-applicant (DAR), in previous research, were adapted during the feasibility phase following an iterative process of qualitative data collection with trainers, CR nurses and patients, combined with lay and clinical advisors’ feedback.

To deliver mental health care coordination, CR nurses will apply clinical decision-making rules based on current best practice [29, 30], matching the intensity of treatment with participant preferences for mental health care. All patients referred to a locality-based CCRP and who agree to undertake an initial assessment (stages 2–3), will be routinely screened for depressive symptoms using the Patient Health Questionnaire (PHQ-9) [58, 59]. This tool assesses the nine *Diagnostic and Statistical Manual of Mental Disorders* (DSM-IV) depression criteria, with individuals scoring 10 or more (out of a possible score of 27) during their initial CR assessment deemed eligible for inclusion.

On identifying an eligible individual, the nurse will explain what evidence-based treatment options are available. This includes BA self-help materials supported by the nurse, GP referral, referral to local IAPT services, and/or referral to specific cardiac patient psychological support services where available. Nurses will coordinate care by monitoring symptoms of depression and anxiety, assessing risk to self or others and agreeing a plan of care with participants that may include BA and/or referral to GP, IAPT or other therapeutic agencies as described above.

As part of this care coordination protocol, all participants will be offered nurse-supported self-help using a participant BA manual. The self-help BA manual consists of a structured programme aimed at enabling participants to re-engage with sources of positive reinforcement from their environment and to develop future strategies for managing their depressive symptoms. The manual adopts a functional analytical approach, aiming to help the participant develop an understanding of behaviours that might interfere with meaningful, goal-oriented behaviours (e.g. negative avoidant behaviours). The manual also explains how to self-monitor mood, and then to identify patterns of behaviour associated with their depression. Participants are encouraged to develop alternative behaviours which are goal- orientated, targeting routine, pleasurable, and necessary activities, and activity scheduling of these identified behaviours.

Although our pilot intervention involves a participant-led self-help BA manual, the CR nurses will actively support participants. The CR nurses will attend a 2-day training course delivered by experienced mental health practitioners, during which they will receive training on the key techniques of mental health care coordination, managing safety and risks, and BA. This training will allow nurses to actively support participants during care coordination and as they work through the BA manual alongside their CR programme. Nurses will also be provided with a session-by-session guide, detailing the types of care coordination tasks which might be relevant for participants as they move through their 6–8 week structured programme (Additional file 1). This guide is flexible to accommodate participant preferences for care. Nurses who are delivering EPC to study participants will also receive bi-weekly clinical supervision by an experienced mental health practitioner.

For those participants who complete CCRP (stage 5), structured details of the care received will be sent to their GP. All participants, including those receiving BA and whose depressive symptoms do not respond, will be given an opportunity to review their management options with the nurse. Here, treatment response is defined as achieving a minimally important clinical difference (MICD) for the PHQ-9 equivalent to a 5-point reduction in score [60]. It is important to note, however, that some people who achieve such a MICD may remain above the PHQ-9 diagnostic threshold (≥ 10). As part of their care coordination, these individuals will be referred for continuing mental health management.

### Randomisation and allocation concealment

An external pilot cluster RCT [56, 57] will be undertaken, seeking to recruit and randomise eight CCRPs (clusters) to a treatment or a control arm in a 5:3 ratio. The randomisation ratio was informed by data emerging from our feasibility study, including the need to ensure that sufficient nurses and patients have EPC exposure to support the aims of the qualitative process evaluation. Randomisation will be conducted using the stratifying variables of CR team setting (hospital-based, community-based, or mixed hospital and community), and monthly throughput of new patients assessed for CR at stages 2–3, facilitating division into small and large teams. Team setting has been selected as a stratifying variable because our feasibility study suggested that teams working in hospital and community settings may encounter different resource issues that could affect the delivery of EPC. Throughput of new patients into CR services has been selected as a stratifying variable, as this is an indicator of team workload, and will ensure that sufficient participants are recruited into each trial arm.

Randomisation will be carried out by a statistician, independent from the researchers recruiting CR teams, using computer-generated random numbers. A cluster randomised design, as opposed to individual participant randomisation, is essential to avoid contamination between trial arms (i.e. intervention participants coming into direct contact with controls and sharing aspects of their treatment). Our EPC intervention involves training CR nurses to apply mental health care coordination with embedded participant-led BA. Once trained, it would be very difficult operationally for the nurse to apply structured care coordination to only a subset of eligible participants (as would be required with individual randomisation).

### Settings and study population

#### CCRP recruitment procedures

Eight locality-based CCRPs in South West England will be recruited and randomised. Teams who took part in the feasibility study will be excluded. A two-stage approach to local CCRP recruitment will be employed. We will write to the lead CR nurse in each of the 20 operationally distinct CCRPs in South West England providing detailed information about the study and inviting their participation. In each of the geographical areas the recruitment letter will be co-signed by the trial chief investigator and the local co-applicant. Each team indicating an interest in participation will receive a briefing visit, where the trial design and methods will be explained by the researcher, and the staff will be given an opportunity to ask questions about what might be involved, before being asked to provide written consent to participate in the study.

Nurses in teams allocated to the CCRP with EPC intervention arm will receive EPC and self-harm risk management training (total of two full days) before they start to recruit patients. EPC-trained nurses will be supervised regularly by experienced specialist clinicians/BA practitioners.

#### Participant eligibility criteria

A pragmatic approach will be adopted, whereby adult patients (aged 18 years or over) referred for CR based on local clinical referral protocols will be screened (stages 2–3) for eligibility. The majority will be individuals previously admitted with an ACS (unstable angina, non-ST-elevation MI or ST-elevation MI) or following a coronary revascularization procedure (percutaneous coronary intervention (PCI), coronary artery bypass grafting (CABG)), with or without heart failure [61]. Patients with a new episode of depressive symptoms identified through nurse screening at the start of CCRP using the PHQ-9 [58, 62] (score 10 or more) are potentially eligible for inclusion. Patient exclusion criteria include: individuals reporting having been actively treated for depression (psychological or drug therapy) within the previous 6 months, or where there is evidence of alcohol or drug dependency, being acutely suicidal, or having poorly controlled bipolar disorder or psychosis/psychotic symptoms based on a clinical review (seeking external confirmation from the GP or other clinicians as required). No language exclusion will be applied; we anticipate that the majority of patients will have sufficiently good English language skills to engage with EPC. However, consistent with routine practice in delivering psychological therapy, NHS translation resources will be employed to assist participants where required.

#### Patient participant recruitment procedure

The CR nurse will apply a structured checklist to ascertain participant eligibility based on our inclusion/exclusion criteria. Eligible participants will be invited to take part in the trial by the CR nurse, and given brief study information to take away and review. The nurse will also ask permission to pass the individual’s contact details on to the study researcher. The researcher will then contact the patient within 7 days to discuss study participation and provide them with a detailed Participant Information Sheet. The researcher will arrange to visit the potential participant to obtain written consent and baseline assessment before the participant commences CCRP (stage 4). Potential participants will be reminded of their right to refuse participation, or to withdraw from the study at any time without it affecting their clinical care.

### Sample size

Using UK national audit data (2010–2011), 55,452 patients (for MI, PCI, CABG) undertook phase 3 CR from 280 teams: an average of 200 patients per team per year. Assuming 17 % have depressive symptoms [27], this equates to 35 eligible patients per team each year. The participant consent rate will be established through piloting as no relevant UK data, applicable to cluster trials, are available. Two RCTs (individual randomisation) from the US, exploring psychological/drug therapy in cardiac rehabilitation settings, observed highly variable consent rates of 85 % (150/177 – Vanguard CODIACS study) [51] and 37 % (2481/6854 – ENRICHD study) [49]. Based on the patient throughput and rates of depressive symptoms observed in our feasibility study, and an assumed participation rate of 50 %, we aim to recruit 64 patients through the eight teams in 6 months. This sample size is sufficient to estimate a follow-up percentage as low as 50 % with margin of error ±12.8 % based on the width of the 95 % confidence interval (CI), and as high as 90 % with a margin of error of ±9.3 % based on the lower bound of the 95 % CI. We do not plan to use estimates from the pilot of the intra-cluster (intra-CR team) correlation coefficients (ICCs) for key outcomes to plan the sample size for the definitive trial as the pilot sample is small and estimates will be imprecise. Using national audit data (2010–2011; 6272 patients; 119 CR teams), we estimate the ICC for depression to be 0.047 (95 % CI: 0.034 to 0.062). This ICC estimate and CI will be used to inform the sample size calculations for the definitive trial.

### Data collection

Participants in both arms will complete baseline measures prior to commencing CCRP, and then two face-to-face follow-up interviews with a researcher at 5 and 8 months post randomisation (Fig. 2). Blinding of patients, practitioners or researchers extracting data on study outcomes is not possible in this cluster design, but the final analysis will be carried out by a statistician who is blind to treatment allocation. Data will be collected on process measures, patient-reported outcomes, and resource use and costs.

#### Process measures

Process data relating to participant throughput and intervention fidelity will be collected.

To ascertain study eligibility and recruitment we will ask CR nurses to report the numbers of patients: attending an initial CCRP nurse assessment, the proportion identified with depressive symptoms during this assessment, the prevalence of ‘new onset’ (as opposed to existing) depression and the number of patients who were eligible for study participation. Of eligible patients, we will describe: the number offered study entry; the number who later agreed to be contacted by a researcher; and who subsequently consented to take part and underwent a baseline interview with a researcher. We will also capture data on participant socio-demographic characteristics (age, sex, ethnicity/preferred language) and clinical condition resulting in a CR referral to document how well we have engaged with service users from diverse backgrounds, such as non-English speakers.

Intervention fidelity will be established through a structured notes review documenting the numbers of participants recruited who do not attend CCRP and the proportion with documentary evidence of care coordination in their notes. For participants engaging with the EPC intervention, we will assess adherence by recording the number of BA sessions offered and the number of sessions attended, the number of participants with documentary evidence of care coordination on exiting CCRP, and the type of psychological referrals made (if appropriate).

#### Participant-reported outcomes measures

Measures are selected on the basis of evidence of their validity in this population, and to ensure coverage of key areas from a clinical and patient perspective (with input from our lay and clinical advisors).

##### Baseline assessment only

This assessment includes some measures that will not be repeated at follow-up. The revised clinical interview schedule (CIS-R) [63, 64] will be administered to ascertain a clinical diagnosis of depression. This score will not determine study eligibility, but will allow its comparison to the other patient-reported measures of depression symptom severity ascertained using the Beck Depression Inventory (BDI-II) (described below). Some short questions to ascertain, and account for, participant treatment preferences [65] will be included. We will extract data from clinical records (GP, CCRP) relating to known cardiac risk factors (body mass index (BMI), blood pressure, glycosylated haemoglobin (HbA1c), lipids, smoking status) as recorded at the participant’s latest clinical assessment prior to recruitment.

##### Baseline and follow-up measures

Although we will request that NHS providers inform us of any participant deaths, we will also tag their NHS records to ensure we capture any cardiac- and non-cardiac-related deaths between baseline and the 8-month follow-up. At the last follow-up we will also request that the date and last recorded known cardiac risk factors in CCRP records are extracted. At the same time we will contact the participant’s GP to request that they extract the same data from primary care records, and that they summarise the incidence of any new cardiac events, i.e. death and/or hospital admissions for ACS or revascularisation procedures (CABG or PCI), or mental health events (e.g. self-harm, suicidality) arising since study enrolment. In addition to being outcomes, these data will be part of the safety monitoring of serious adverse events (SAEs) and adverse events (AEs).

The baseline and follow-up interviews will include self-reported cardiac- and non-cardiac- related morbidity and smoking status, antidepressant medication use [66], and resource use. Depressive symptoms will be measured using the BDI-II [67, 68], a 21-item self-report instrument measuring symptom severity with an emphasis on affective and cognitive symptoms. Higher scores represent greater depression severity (range 0–63), and minimal (0–13), mild (14–19), moderate (20–28) and severe (29–63) symptom severity ranges have been specified. The Beck Anxiety Inventory [69, 70] will be applied as anxiety is commonly comorbid with depression. This 21-item, self-report measure asks patients to report anxiety symptoms over the last week from 0 (none: it did not bother me at all), 1 (mildly: it did not bother me much), 2 (moderately: it was very unpleasant, but I could stand it), and 3 (severely: I could barely stand it). Item scores are summed with total scores of 8–15, 26–25, and 26–63 taken as cut-off points defining categories for mild, moderate, or severe anxiety respectively. Health-related quality of life will be assessed using both generic and disease-specific measures. The 5-item EuroQoL (EQ-5D) [71] is a standardised generic measure of health-related quality of life, which is suitable for use in people with a wide range of health conditions, and is recommended by NICE for economic evaluations alongside clinical trials. We will also administer the HeartQoL [72, 73], a tool comprising 14 items, with 10-item physical and 4-item emotional subscales, which are scored from 0 (poor health-related quality of life) to 3 (better). To assess when and how participants become activated over the course of their EPC, we will administer the 9-item Behavioral Activation for Depression Scale – Short Form (BADS-SF) [74] at baseline, and the 5-month and 8-month follow-up assessments. The BADS-SF is a 9-item tool, where each item is rated from 0 (not at all) to 6 (completely). The total score ranges from 0 to 54, with high scores representing greater activation. Finally, participant experiences of care will be assessed at the 5-month follow-up only, using the 8-item, self-reported Client Satisfaction Questionnaire (CSQ-8) [75] and an adapted version of the NHS Friends and Family Test (FFT). The CSQ items use a 4-point Likert scale (1 to 4) response set, with the total scores ranging from a possible 8 to 32; and higher values indicating greater satisfaction. The FFT consists of a single item with a 5-point rating scale (‘Extremely likely’ to ‘Extremely unlikely’) and two free-text items (‘What was good about your experience?’ and ‘What would have made your experience better?’).

### Economic evaluation

The analytical perspective of NICE’s decision-making is a societal costing perspective. The pilot study will test methods of resource and service use data collection enabling estimation of the costs to the NHS, costs to social care and PSS, and relevant costs to patients and their carers/families (including NHS and privately funded mental health care). Wherever feasible, these will distinguish use of services for (1) depression or anxiety; (2) for other mental health problems; (3) for other health reasons or social needs. A preliminary assessment of the cost of providing EPC within CR will be undertaken. Service use data will be collected from routine/administrative sources (e.g. hospital records, GP records, community mental health team records, social care records) and participant self-report using the Service and Resource Use Questionnaire (SRUQ). The SRUQ will be adapted from the Client Services Receipt Inventory [76], with input from our lay advisors. The completeness, validity and reliability of participant-reported versus routine administrative data will be compared in relation to the types/amounts of health care/service used over the follow-up periods planned for a definitive trial.

#### Statistical analysis plan

Trial data will estimate CR team recruitment and patient study completion rates. As this is a pilot study we will not report definitive estimates of effectiveness and costs; our primary aim is to document the adequacy of trial procedures, intervention acceptability and outcome measures. All pilot study data will be collected [77] and reported according to the CONSORT extension for cluster randomised trials [78]. Recruitment, intervention and control uptake, outcome completion rates, and drop out will be reported (with 95 % CIs). We will compare the recruitment rate and participant characteristics at baseline between the trial arms to assess whether there is evidence of selection bias resulting from (cluster) randomising teams before participant recruitment [79]. Patient outcome data (including resource use) will be analysed descriptively (e.g. means, standard deviations (SDs)) for each arm at each follow-up and we will report outcome effect sizes (i.e. between-group mean differences and 95 % CIs) based on an intention-to-treat analysis. We will explore the impact of patient preferences documented at baseline in the analysis. We will also conduct an exploratory sensitivity analysis reporting between-group outcomes in the sub-groups of patients who do, or do not attend, cardiac rehabilitation programmes.

## Qualitative interview study

### Data collection

Several methods of data collection will be used. The researcher will observe staff training to identify what issues nurses appear to struggle with and what practical concerns they have about EPC. Observations will be recorded through extensive field notes. Nurses trained in EPC will be invited to take part in an interview towards the end of EPC delivery (once they have gained experience in delivering the intervention), to explore their views on the nurse manual, and to assess their views and experiences of delivering EPC within existing programmes. These interviews will be held by an experienced qualitative interviewer (RW) following a topic guide. Interviews will be conducted either by telephone or on a face-to-face basis, as the teams will be based across a large geographical area and telephone interviews can be used to gather the same material as those held face-to-face [80]. Written consent will be secured from those interviewed in person and verbal consent will be gained from those interviewed by telephone. It is predicted that up to 15 nurses will be involved in delivery of EPC in the five CCRPs allocated to the intervention arm. The aim will be to interview around 10 of these nurses.

In-depth interviews will be conducted with patients recruited to the trial who subsequently agree to take part in qualitative interviews. When sampling these patients we will aim for maximum variation in relation to trial arm, age, CR team, gender, socio-economic background, depression score at baseline and adherence to the intervention. Participants will be interviewed shortly after they have completed their 5-month follow-up measurements in case the interview process influences their views of the intervention or trial. Participants will be given an opportunity to ask questions before the interview takes places, and written informed consent will be obtained prior to commencing. All interviews will be conducted by RW following a topic guide. Individuals will be interviewed by telephone or on a face-to-face basis, and verbal or written consent secured respectively. It is predicted that about 30 participants recruited to the trial will be interviewed (10 usual care, 12 completers and 8 non-completers of EPC).

Interviews will also be held with patients who declined to take part in the trial to identify reasons for their decision. These interviews will usually be held by telephone to encourage participation. The data gathered will indicate ways to maximise recruitment and to reflect upon how representative individuals in the trial are of other CR patients. About 10 ‘decliners’ will be interviewed early on in the trial.

### Qualitative analysis plan

Data collection and analysis will proceed in parallel. All interview data will be audio-recorded, transcribed verbatim and analysed thematically with the aid of NVivo, and using an approach based on a framework approach [81]. Analysing the data thematically will enable comparisons to be made within and across the interviews, and for the views of staff and patients to be compared and contrasted regarding specific topics, such as intervention acceptability.

## Ethical and governance considerations

The Royal Devon and Exeter NHS Foundation Trust is acting as the trial sponsor. This paper describes the CADENCE study protocol (version 4, 19 June 2015), which was reviewed and a favourable ethical opinion obtained from the National Research Ethics Service (NRES) Committee South West – Exeter (reference: 14/SW/0139) and the relevant NHS research governance approvals were obtained prior to commencing fieldwork from: the Royal Cornwall Hospitals NHS Trust; Somerset Partnership NHS Foundation Trust; Taunton and Somerset NHS Foundation Trust; Torbay and South Devon NHS Foundation Trust; University Hospitals Bristol NHS Foundation Trust; University Hospitals Coventry and Warwickshire NHS Trust; Weston Area Health Trust; and Yeovil District Hospital NHS Foundation Trust. Any protocol amendments will be notified through existing pathways in the UK NRES and NHS research governances system, and the trial team will ensure that all participating CCRPs, staff and patients, and trial registries are informed of any changes to the protocol.

This study is registered with a trials registry (ISRCTN34701576) and has been adopted by the UK Comprehensive Research Network (UKCRN ID: 17105). All researchers and nurses involved in participant recruitment will undertake Good Clinical Practice (GCP) training.

### Data management

All personal information obtained about patients or staff for the purposes of recruitment or data collection will be held in accordance with the UK Data Protection Act, 1998. Each participant will be assigned a research number and all outcome data will be encrypted and stored without the participant’s name or address. Electronic study records will be stored in a SQL server database managed by the Peninsula Clinical Trial Unit (PenCTU), hosted on a secure server maintained by Plymouth University. Access to electronic data will be permission-based, with access to identifiable information limited to those processing questionnaires and performing initial screening activities. Copies of study data retained at participating sites will be securely stored for the duration of the study prior to archiving. Electronic copies of any letters or e-mails sent to study participants will be stored securely on a password-protected computer at the University of Exeter, and paper-based information held in a locked filing cabinet in the research team offices. Names and participant details will not be passed onto any third parties and no named individuals will be included in the write-up of the results. The only time personal information would be passed on to a third party (i.e. the patient’s GP) would be if we considered there to be risk of serious harm to a research participant and safety protocols need to be activated.

All study data will be kept for 10 years under secure conditions on University of Plymouth and University of Exeter servers. We will seek consent from participants for core outcome data from the pilot study to be released in an anonymised form to an independent data repository (for example, the UK Data Archive) following publication of the key planned outcome papers.

### Safety of participants and researchers

While we believe the study has minimal implications for health care staff, participants will have been found to have depressive symptoms and it is, therefore, appropriate to have a safety policy in place regarding the management of potentially serious adverse events (e.g. self-injury or suicidality). The Participant Information Sheet will let the person know that if we are very concerned about their safety or someone else’s safety we may need to break confidentiality and inform their GP.

Involvement in research interviews where participants are asked to reflect on their health issues can, in some cases, cause distress. Should any such difficulties occur during a research interview, the researcher will offer support to the person involved. Should a participant appear significantly upset and at risk as a result of the interview, the researcher will (with permission) advise the individual’s GP of this distress and encourage the person to seek further support from the support network available to them. Should there be any concern that a participant is likely to cause harm either to themselves or another person, their GP will be notified immediately by a senior clinician from the study team. Since researchers will be conducting interviews alone with participants, potentially in the participant’s own home, it will be important to have safeguards in place to protect both participant and researcher. Disclosure and Barring Service checks will be performed on any researcher taking part to ensure that they are an appropriate person to be working with vulnerable adults. To ensure the safety of researcher and patient, a lone-worker policy will be applied, providing a mechanism for ensuring that the exact whereabouts of researchers and patients engaged in research activities is known.

## Patient and public involvement

Patient and public involvement (PPI) has been sought during protocol development. The NIHR CLAHRC for the South West Peninsula (PenCLAHRC) hosts the Peninsula PPI Group (http://clahrc-peninsula.nihr.ac.uk/patient-and-public-involvement-in-research). This group has published a framework regarding PPI involvement in research [82], and provides research training and support to facilitate the public becoming actively involved in all aspects of the research process. The co-applicant (AG, PPI facilitator) recruited four lay advisors with relevant, lived experience, each of whom reviewed and commented on our research proposal. PPI will continue throughout this project, with the precise roles of advisors negotiated across time. In the early stage, our lay advisors reviewed outcome measures and informed the design of all study-related materials, e.g. information sheets and consent forms. Our lay advisors were also involved in the development of the intervention and accompanying training manual. This helped ensure the acceptability of the intervention to patients. Four lay advisors also attend our project management meetings on a quarterly basis and actively participate in decisions related to the ongoing running of the project. A key area of future involvement will be in dissemination plans. Our lay advisors will work with us to proactively shape the BACPR standards, ensuring that the needs of patients with depression are embedded within the core service descriptions. When sitting on committees, lay advisors will have equal voting rights compared with academic/clinical members. Lay advisors will be paid costs for travel and for time spent at meetings in line with INVOLVE guidance on good practice. AG will support lay advisors attending meetings and/or identify appropriate training opportunities. This will maximise the ability of our lay representatives to actively contribute to our work.

## Project oversight

JC (chief investigator) and SR (scientific lead) will supervise and oversee the strategic development and progress of the project, liaising with the wider team and methodological leads on a routine basis. All research fellows will be based in Exeter (with JC and SR) and will manage the day-to-day aspects of the research relevant to their own role, supported by an administrator at peak periods of fieldwork activity. KT will provide qualitative expertise and supervision, RA is the health economics lead, OCU is the statistics lead, and AG will lead PPI engagement. Monthly project management meetings will be organised to ensure co-applicants and two lay advisors are kept informed of progress and contribute to the ongoing steering of the research.

As this is a pilot trial, a data monitoring committee has not been convened. A Trial Steering Committee (TSC) with independent chair will be appointed in accordance with MRC guidelines. Members will represent an appropriate range of perspectives and expertise (e.g. two lay advisors, clinicians, methodologists), meeting at critical points in the project timeline. The TSC will monitor the scientific quality, ethics and general progress of the project, and will monitor the SAE/AE data emerging from the study.

## Project timeline

The project commenced on 1 April 2014 and is scheduled to complete in July 2016. The feasibility study was reported in May 2015. Preparatory work for the pilot trial, including CR team recruitment, commenced January 2015. Cluster randomisation took place in April 2015, and intervention training delivered to the five CR teams in June–July 2015. Participant recruitment commenced July 2015 and is scheduled to complete by the end of 2015. Follow-up data and qualitative interviews will take place between September 2015 and June 2016. Data analysis will commence in January 2016, with the final report due in July 2016.

## Discussion

This paper presents the study protocol (version 4, 19 June 2015) for the CADENCE study, reported to satisfy the Standard Protocol items: Recommendations for Interventional Trials (SPIRIT) guidelines (Additional file 2). This study will provide important information supporting the development and subsequent evaluation of psychological treatment for patients identified with depressive symptoms following an ACS or revascularisation procedure. Our EPC intervention involves training CR nurses in the application of evidence-based mental health care co-ordination algorithms, and in how to actively support and monitor patients working through a BA self-help manual with associated tasks. While both mental health care co-ordination and BA have previously been demonstrated to be effective treatments for patients with depression, their application to busy CR settings and patients is untested. A crucial element of this research will be to examine the feasibility and acceptability of implementing mental health care management strategies within the routine care. Study outcomes will inform the design of a definitive clinical trial that will provide evidence of the effectiveness, cost-effectiveness and acceptability of embedding psychological care within the CR care pathway.

This study was funded in response to a commissioning brief from the Health Technology Assessment board of the UK the National Institute for Health Research (NIHR; project reference 12/189/06), which identified this research area as an important knowledge gap and which sought to investigate an intervention embedded within routine care. We will disseminate our findings widely, to ensure this research will guide policy-makers and clinicians in the future development and organisation of psychological care for patients with depressive symptoms engaging with CR services. We anticipate that data on acceptability will be of immediate interest to patients and their families, professionals responsible for the design and implementation of CR services, and to the professional body (BACPR) responsible for defining core services and standards of care within NHS CR services.

## Trial status

Patient participant recruitment commenced in July 2015 and is ongoing, scheduled for completion in December 2015.

## Additional files

Additional file 1:
**Guide for session planning for nurses delivering enhanced psychological care as part of a comprehensive cardiac rehabilitation programme.** (DOCX 44 kb) Additional file 2:
**SPIRIT Checklist: CADENCE study protocol (version 4, 19 June 2015).** (DOCX 61 kb)

## Abbreviations

ACS: acute coronary syndrome
ADM: antidepressant medication
AE: adverse event
BA: behavioural activation
BACPR: British Association for Cardiovascular Prevention and Rehabilitation
BADS-SF: Behavioral Activation for Depression Scale – Short Form
BDI-II: Beck Depression Inventory
BMI: body mass index
CABG: coronary artery bypass grafting
CBT: cognitive behavioural therapy
CIS-R: revised clinical interview schedule
CR: cardiac rehabilitation
CRRP: comprehensive cardiac rehabilitation programme
CSQ-8: Client Satisfaction Questionnaire (CSQ-8)
EQ-5D: EuroQoL Index
FFT: Friends and Family Test
GP: general practitioner
IAPT: Improving Access to Psychological Therapies
ICC: intra-cluster correlation coefficient
MI: myocardial infarct
MICD: minimally important clinical difference
NHS: National Health Service
NICE: National Institute of Clinical Excellence
NIHR: National Institute for Health Research
PCI: percutaneous coronary intervention
PHQ-9: Patient Health Questionnaire-9
PPI: patient and public involvement
SAE: serious adverse event
SRUQ: Service and Resource Use Questionnaire
