# Supplementary material for: Assessing the effectiveness of enhanced psychological care for patients with depressive symptoms attending cardiac rehabilitation compared with treatment as usual (CADENCE): study protocol for a pilot cluster randomised controlled trial
Source: Trials. 2016 Feb 2;17:59. doi: 10.1186/s13063-016-1184-9 (PMC4736496; doi:10.1186/s13063-016-1184-9)
Supplement: Additional file 1: — Guide for session planning for nurses delivering enhanced psychological care as part of a comprehensive cardiac rehabilitation programme. (DOCX 44 kb) [file 13063_2016_1184_MOESM1_ESM.docx]

**Additional file 1:** Guide for session planning for nurses delivering enhanced psychological care as part of a comprehensive cardiac rehabilitation programme

| **First CCRP session:** Explain to participant the options available i.e. supported self-help BA manual, with or without onward referral to relevant mental health care services, depending on patient preferences.  Agree mental health treatment plan and take relevant action. This might include:   - agreeing to discuss the self-help BA book next time you meet (or arranging a special follow up telephone appointment) - writing to or telephoning the patient’s GP - giving out details of the local IAPT service or making a referral yourself - arranging a specialist cardiac psychological therapy referral   **All remaining CCRP sessions:** Content tailored depending on whether the participant has decided to follow self-help BA manual. These sessions can be brief depending on patient progress.  *Care co-ordination only*. Review mood since last appointment, using PHQ-9 and GAD-7 if preferred. Check that the patient’s mood is not deteriorating further, that they are safe and to see if the action you agreed with the patient to take has been followed up (i.e. did the participant make the GP appointment?).  *Self-help BA manual.* At each session assess symptoms and risk, review treatment choices, support BA, and future planning. BA support is aimed at helping participants to engage with the self-help manual, explaining ideas and methods as required.  At **the mind point of CCRP (e.g. around 4 weeks)** dedicated clinic time should be allocated to reviewing progress and carefully review treatment options.  At the **final CCRP session (e.g. around week 6-8)** dedicated time should be allocated to reviewing progress, and structured details of the care received will be sent to their GP. Participants who fail to respond to self-help BA will be referred on to their preferred management option. |
| --- |
